# Ready to learn: goal orientation and academic performance in a service-learning course for health professions students

**DOI:** 10.3389/fpsyg.2026.1880789

**Published:** 2026-09-30

**Authors:** Kathleen N. Heeter, Jonathan T. Macy, Angela Chow, Corey Kalbaugh, Samantha C. Schaefer, Lilian Golzarri-Arroyo, Kyle A. Kercher, Vanessa M. Martinez Kercher

**Affiliations:** 1 Department of Applied Health Science, School of Public Health-Bloomington, Indiana University, Bloomington, IN, United States; 2 Department of Epidemiology and Biostatistics, School of Public Health-Bloomington, Indiana University, Bloomington, IN, United States; 3 Healthy IU (Workplace Wellness Program), Indiana University Human Resources, Bloomington, IN, United States; 4 Department of Kinesiology, School of Public Health-Bloomington, Indiana University, Bloomington, IN, United States

**Keywords:** academic self-efficacy, experiential learning, goal orientation, health professions education, service-learning

## Abstract

**Introduction:**

Goal orientation and academic self-efficacy are central constructs in educational psychology; however, their roles in applied, interpersonal learning environments remain underexplored. This study examined relationships among mastery, performance, and avoidant goal orientations, academic self-efficacy, and academic performance in a service-learning course for health professions students. The mediating role of academic self-efficacy was also evaluated.

**Methods:**

Seventy-four undergraduate and graduate public health students enrolled in a semester-long wellness coaching service-learning course completed baseline measures of goal orientation and academic self-efficacy. Academic performance was assessed using final course grades. Pearson correlations and mediation analyses were conducted while controlling for age and gender. Indirect effects were estimated using bootstrapped confidence intervals with 5,000 resamples.

**Results:**

Performance and avoidant goal orientations were positively associated with academic performance, whereas mastery goal orientation was not significantly associated with academic outcomes. Mastery and performance goal orientations were positively associated with academic self-efficacy; however, academic self-efficacy was not significantly associated with academic performance and did not mediate the relationship between goal orientation and academic performance.

**Discussion:**

These findings differ from patterns commonly observed in traditional academic settings, where mastery orientation and academic self-efficacy are typically stronger predictors of achievement. Results suggest that the influence of motivational orientations may depend on learning context, particularly in environments emphasizing interpersonal performance and applied skill use. These findings highlight the importance of considering contextual factors when applying motivational theory to experiential learning settings.

## Introduction

Understanding how motivational processes shape learning and performance is a central concern in educational psychology. These processes are particularly important in fields such as public health, where students must not only acquire knowledge but also develop the skills needed to apply that knowledge in real-world settings. A growing number of public health students are entering healthcare and social assistance professions (e.g., nursing, medicine, public health practice), highlighting the need to understand how students learn to effectively translate knowledge into practice (Leider et al., 2023). How and why students learn is especially critical in these contexts, as learning processes influence problem-solving, motivation, and persistence (van Dierendonck and van der Gaast, 2013; Kaplan and Maehr, 2007). Many of these students will engage directly in patient care, making it essential to understand the factors that support effective learning and skill development.

Goal orientation (GO) theory conceptualizes individuals’ reasons for engaging in achievement-related tasks and has been widely used to explain variation in academic performance. Rather than focusing only on ability, GO emphasizes the purposes behind engagement in learning tasks, and in turn the cognitive, behavioral, and emotional responses to such academic challenges (Honicke and Broadbent, 2016; Ng and Bereiter, 1991). The three primary orientations identified in GO theory are mastery, performance, and avoidant (Stavropoulou and Stamovlasis, 2025). Mastery orientation reflects a focus on developing competence and understanding, often associated with persistence and deep learning strategies (Stavropoulou and Stamovlasis, 2025). Performance orientation emphasizes demonstrating competence relative to others, which may promote effort when success is easily attainable but can also lead to surface-level learning strategies (Stavropoulou and Stamovlasis, 2025). Avoidant orientation involves a focus on avoiding negative judgments or failure, and is often linked to disengagement or maladaptive learning behaviors (Pintrich, 2000).

Research examining GO has mixed findings regarding its relationship with academic performance. Mastery GO has been associated with positive outcomes such as deeper engagement and higher achievement; however, these effects have not consistently been observed across multiple contexts (Lin et al., 2009). Performance GO has been found to have both positive and negative academic outcomes, depending on the learning environment and evaluation structure used (Pintrich, 2000; Alrakaf et al., 2014). Avoidant GO has historically been associated with poorer academic outcomes; however, its negative effects may vary in contexts where performance is socially evaluated (Pintrich, 2000; Artino, 2012; Laitinen et al., 2024; Eum and Rice, 2011). These inconsistencies suggest that the relationship between GO and academic performance may be context-dependent, and that findings from traditional classroom settings may not fully generalize to applied or interpersonal learning environments.

Academic self-efficacy (Academic SE), derived from social cognitive theory, refers to individuals’ beliefs in their capacity to successfully organize and execute the actions required to achieve desired academic outcomes (Artino, 2012; Schunk, 1991; Jamali et al., 2013). These beliefs can play a central role in regulating academic behavior, as individuals with higher academic SE are more likely to exert effort, persist during challenges, and engage in effective learning strategies, which all positively contribute to academic performance (Jamali et al., 2013; Matovu, 2020). Research has consistently found positive associations between academic SE and academic outcomes such as increased grade point average and course performance among university students (Jamali et al., 2013; Matovu, 2020).

Achievement Goal Theory and Social Cognitive Theory provide a theoretical basis for the proposed mediating role of academic SE. Achievement Goal Theory suggests that students’ GOs influence how they define competence and interpret their achievement-related experiences, such as challenges and feedback. Students with mastery GO tend to evaluate competence in terms of learning and improvement, whereas students with performance GO focus on demonstrating competence relative to others, and students with avoidant GO focus on avoiding failure or negative judgments (Pintrich, 2000). These differing interpretations of achievement may shape students’ beliefs about their capability to successfully perform academic tasks. Additionally, Social Cognitive Theory posits that these beliefs about capability are reflected in academic SE, which can influence effort, persistence, and overall responses to academic challenges (Artino, 2012; Schunk, 1991). Thus, GO may influence how students interpret achievement experiences, which may shape academic self-efficacy and, in turn, academic performance, providing the rationale for examining academic SE as a potential mediator between GO and academic performance (Honicke and Broadbent, 2016).

Prior research provides empirical support for examining this pathway, suggesting that academic self-efficacy may mediate the relationship between goal orientation and academic performance, particularly for mastery and performance orientations (Honicke and Broadbent, 2016; Honicke et al., 2020). However, these relationships have primarily been examined in traditional academic settings and often exclude avoidant GO, limiting understanding of how these constructs interact across different learning contexts.

Despite substantial research examining GO and academic SE, limitations remain in understanding how these constructs interact to influence academic performance. Most existing literature involves studies conducted in traditional classroom environments that utilize test- or individual assignment-based performance criteria. In these settings, evaluation is often discrete and task-based, with performance communicated through grades or instructor feedback. In contrast, authentic and applied assessments in health professions education require students to demonstrate skills in contexts that more closely approximate real-world practice (Jones et al., 2024). Service-learning further situates performance within interpersonal environments in which students’ behaviors and competencies may be directly observed by community partners, peers, or instructors over the course of the experience (Roth et al., 2025). Evaluation may therefore be experienced not only as an academic judgment of a completed task but also as an ongoing interpersonal evaluation of competence during performance. This structural difference may increase the salience of demonstrating competence or avoiding negative judgment and, consequently, alter how goal orientations are expressed behaviorally (Pintrich, 2000; Senko and Harackiewicz, 2002). Thus, relationships among motivational orientations, beliefs about competence, and academic performance established in traditional classroom settings may not fully generalize to applied or interpersonal learning environments.

As a result, it remains unclear whether the previously established relationships among GO, academic SE and academic performance generalize into a service-learning setting. Additionally, prior research has often excluded avoidant GO or examined it separately, limiting a comprehensive understanding of how all three GO subtypes operate together. Furthermore, although academic SE has been a proposed mediator between GO and academic performance, this relationship has not been consistently tested across different learning environments. Addressing these gaps is necessary to refine theoretical models of achievement motivation and to better understand how motivational processes operate in applied learning contexts.

This study addresses these limitations by examining the relationships among GO subtypes, academic SE and academic performance within an applied service-learning course called Ready to Move (RTM) at a large public midwestern university. By including all three GO subtypes and testing the mediating role of academic SE, this study endeavors to provide a more comprehensive evaluation of how motivation constructs can interact to influence performance. Additionally, this work extends GO theory by assessing whether established relationships among these variables hold in learning contexts that emphasize interpersonal performance and experiential skill application and, in doing so, contributes to a more nuanced understanding of how achievement motivation operates across different educational environments.

The aim of the study was to examine the relationships among GO subtypes, academic SE, and academic performance in an applied service-learning course. Based on prior goal orientation research, we hypothesized that (H1a) mastery GO would be positively associated with academic performance, (H1b) performance GO would be positively associated with academic performance, and (H1c) avoidant GO would be negatively associated with academic performance.

We further hypothesized that academic SE would mediate these relationships, such that (H2a) mastery GO and (H2b) performance GO would be associated with higher academic SE and, in turn, higher academic performance, whereas (H2c) avoidant GO would be associated with lower academic SE and, in turn, lower academic performance.

## Materials and methods

This study used a prospective observational design to examine relationships among GO, academic self-efficacy, and academic performance. Participants were recruited using convenience sampling from a university-based service-learning course. The sample size was determined by the number of eligible students enrolled in the service-learning course during the two study semesters; therefore, an *a priori* power analysis was not conducted. Eligible participants included third- and fourth-year undergraduate and graduate students enrolled in kinesiology-based public health courses who consented to participate. Students’ majors included exercise science, physical activity, and fitness and wellness. No exclusion criteria were applied.

The study received an exempt determination from the university’s Institutional Review Board (IRB #12340), and informed consent was obtained from all participants.

### Service-learning course

A total of 74 students participated in a 16-week service-learning course that focused on health and wellness coaching over two semesters. This course represents an applied, service-learning environment in which student performance is evaluated through both knowledge acquisition and real-world coaching interactions with clients, rather than solely through traditional examinations. Figure 1 depicts the components of the service-learning course.

**Figure 1:**
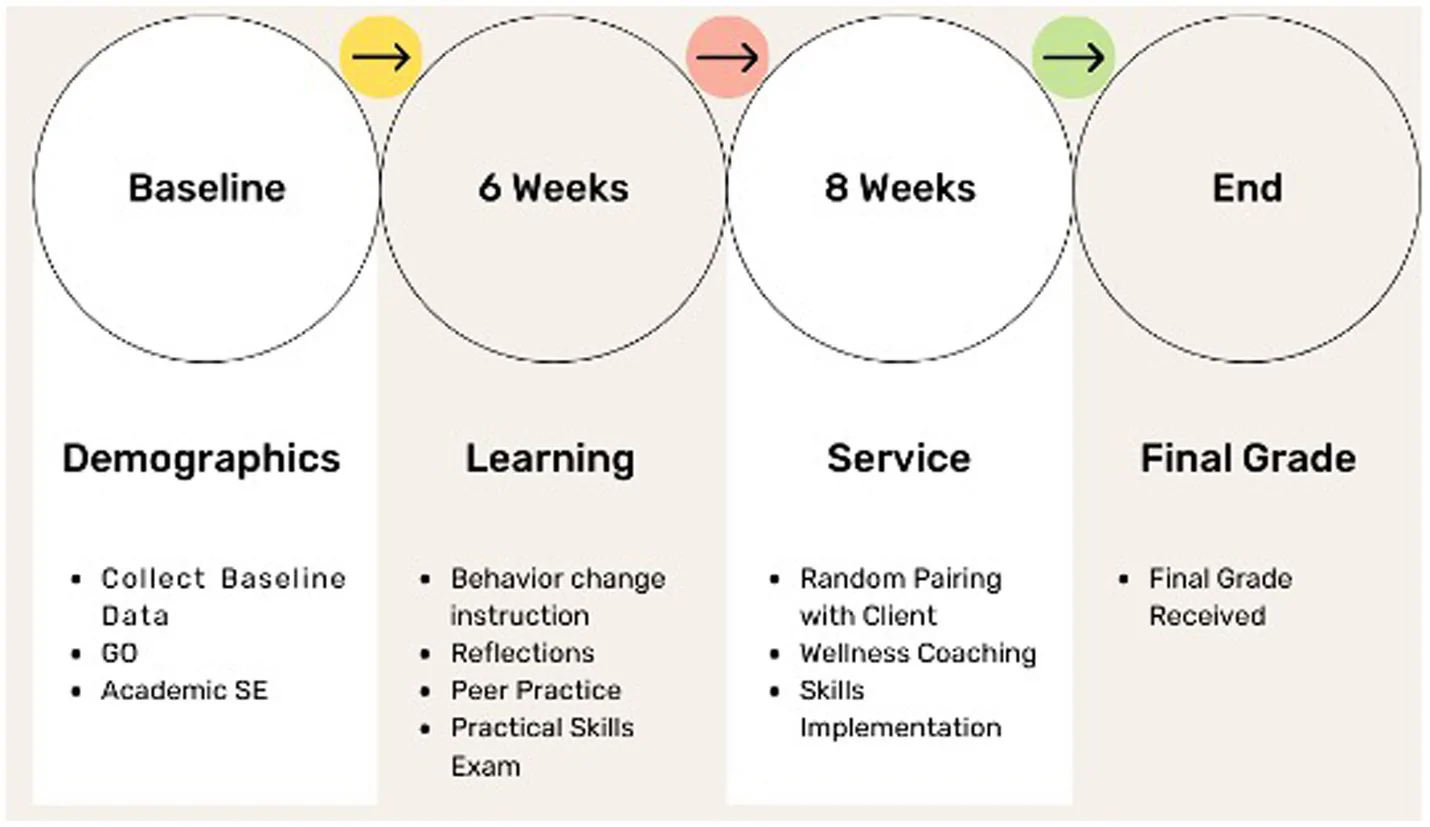
Service-learning course flow.

#### Course instruction

The first 6 weeks of the course focused on student training. During this time, students gained foundational knowledge of behavior-change strategies, including motivational interviewing (mindful listening, perceptive reflections, and open-ended inquiry), goal setting, and program development. This education included in-class activities where students could practice and apply their coaching techniques with multiple peers. At the end of the 6-week intensive training, all students completed a practical skills assessment in which they applied foundational coaching skills with a mock client. Both cohorts of classes were taught by the same professor, using the same syllabus, assessment procedures, and service-learning procedures.

The sample included 64 undergraduate students (28 Cohort 1, 36 Cohort 2) and 10 graduate students (5 Cohort 1, 5 Cohort 2), all of whom were public health majors. Race and ethnicity were not collected from study participants; however, the race and ethnicity demographics of the University are 68.6% White, 7.9% Hispanic, 7.4% Asian, and less than 5% Black or African-American (Factual, 2025). An exploratory survey item assessed prior coaching experience; however, responses reflected a broad range of experiences, some of which could not be clearly classified as coaching. Because the item did not adequately distinguish relevant prior coaching experience, it was not included as a covariate in the analyses.

#### Service-learning

Following the intensive training portion of the course, each student was randomly paired with a university employee (staff or faculty member) for 8 weeks as health and wellness coaches in a program called Ready to Move, in collaboration with the university’s workplace wellness program. The program, which provides participants with weekly coaching sessions and communications with their student coaches, focuses on building healthier habits in their lives.

### Measures

GO and academic SE were assessed at baseline, and academic performance was measured following course completion using final course grades.

#### Goal orientation

The Goal Orientation Scale (GO-15) from Patterns of Adaptive Learning Skills was used to assess GO at baseline. The 15-item questionnaire consists of three subscales measuring students’ perceptions of their motivation to complete classwork. Items were rated on a 5-point Likert scale ranging from 1 = ‘not true at all’ to 5 = ‘very true.’ Scores for each GO subscale were calculated as the mean of items within each subscale, with higher scores indicating greater endorsement of each GO. The GO-15 has been validated among emerging adults, with reported Cronbach’s alpha values ranging from 0.75 to 0.85 (Anderman et al., 2005).

#### Academic self-efficacy

The Academic Self-Efficacy Scale (ASES-5) is a 5-item Likert-type scale used to assess students’ perceptions of their competence to do their course work at baseline. The responses ranged from 1 = “not true at all” to 5 = “very true.” Scores were calculated as the mean across items, with higher scores indicating higher SE. The scale has been used to assess academic SE across student populations, including college students, with a reported Cronbach’s alpha of 0.78 (Nielsen et al., 2018).

#### Academic performance

Final course grade was used as a measure of academic performance. Final course grade was calculated as the percentage of points earned relative to the potential points available for the course. Course grades were derived from standardized assessment components evaluated by the same instructor using a consistent rubric. Grade assessment components are shown in Table 1.

**Table 1:** Assessment methods and total points for service-learning course.

Assessment methods	Total points	Grade %
Content checks quizzes	80	13.30%
Content reflection	200	33.30%
Coaching practical skills	100	16.60%
Coaching presentations	100	16.60%
Coaching check-ins	120	20%
Total points	600	100%

### Analysis

Baseline data were collected using Qualtrics (Qualtrics, Provo, UT), and final course grades were obtained from the course instructor. Data were analyzed using RStudio (version 2024.12.1 + 563; RStudio, Boston, MA). Prior to the analysis, data were screened for missing values, outliers, and normality; no missing data were identified. Descriptive statistics were calculated for all continuous variables, and frequencies were calculated for categorical variables.

Pearson correlations were conducted to examine relationships among GO subscales, academic self-efficacy, and academic performance.

To examine mediation, three separate models were tested, with each GO subtype (mastery, performance, avoidant) entered as the predictor, academic self-efficacy as the mediator, and academic performance as the outcome. In each mediation model, c′ represents the direct effect of GO on academic performance after accounting for the indirect pathway through academic SE. All models controlled for age and gender because these demographic characteristics have been associated with variation in academic performance and academic SE (Huang, 2013; Sheard, 2009). Prior coaching experience was not included as a covariate because the exploratory survey item did not permit consistent classification of relevant coaching experience, as described above. Indirect effects were estimated using bootstrapped confidence intervals with 5,000 resamples. An indirect effect was considered statistically significant if the 95% bootstrap confidence interval did not include zero.

## Results

Descriptive statistics are presented in Table 2. Among the 74 students included in the study, 86.5% were undergraduate students, and gender was relatively evenly distributed (36 men [48.6%] and 38 women [51.4%]). The mean age of participants was 21.6 years. Among GO subscales, mastery GO had the highest mean score, followed by avoidant and performance orientations. The mean academic self-efficacy score was 4.51 on a 5-point Likert scale. The mean final course score was 86.5%, with scores ranging from 64.5 to 100%.

**Table 2:** Ready to move study descriptive statistics.

Variable	M	SD	Min	Max
Goal orientation (GO)				
GO Mastery	4.61	0.51	3.00	5.00
GO Performance	2.38	0.90	1.00	4.80
GO Avoidant	3.08	1.00	1.00	4.80
Academics				
Academic self-efficacy	4.51	0.52	3.00	5.00
Final score	86.50	9.41	64.50	100.00
Demographics				
Age	21.60	3.33	19.00	40.00

M, Mean; SD, Standard Deviation; Min, Minimum; Max, Maximum; GO, Goal Orientation represented by subscales mastery, performance, and avoidant. SE, self-efficacy.

Performance GO (*r* = 0.30, *p* < 0.001) and avoidant GO (*r* = 0.30, *p* < 0.001) were both significantly and positively correlated with academic performance. Notably, the magnitude of the association between avoidant GO and academic performance was similar to that observed for performance GO, despite avoidant GO typically being associated with poorer academic outcomes. In contrast, mastery GO was weakly and not significantly associated with academic performance (*r* = 0.21, *p* > 0.05). Mastery GO (*r* = 0.52, *p* < 0.001) and performance GO (*r* = 0.27, *p* < 0.05) were significantly and positively correlated with academic self-efficacy, whereas academic self-efficacy was not significantly associated with academic performance (*r* = 0.10, *p* > 0.05). Thus, H1b was supported, whereas H1a was not supported and H1c was not supported, with avoidant GO demonstrating an association in the opposite direction from that hypothesized.

Across all three models, the indirect effects of goal orientation on academic performance through academic SE were not statistically significant. The indirect effect was 0.345 (95% bootstrap CI [−2.416, 3.188]) for mastery GO, 0.142 (95% bootstrap CI [−0.503, 1.013]) for performance GO, and 0.138 (95% bootstrap CI [−0.336, 0.951]) for avoidant GO. Because all confidence intervals included zero, academic SE did not significantly mediate the relationship between any GO subtype and academic performance. Results of these models are shown in Figure 2. Accordingly, H2a–H2c were not supported.

**Figure 2:**
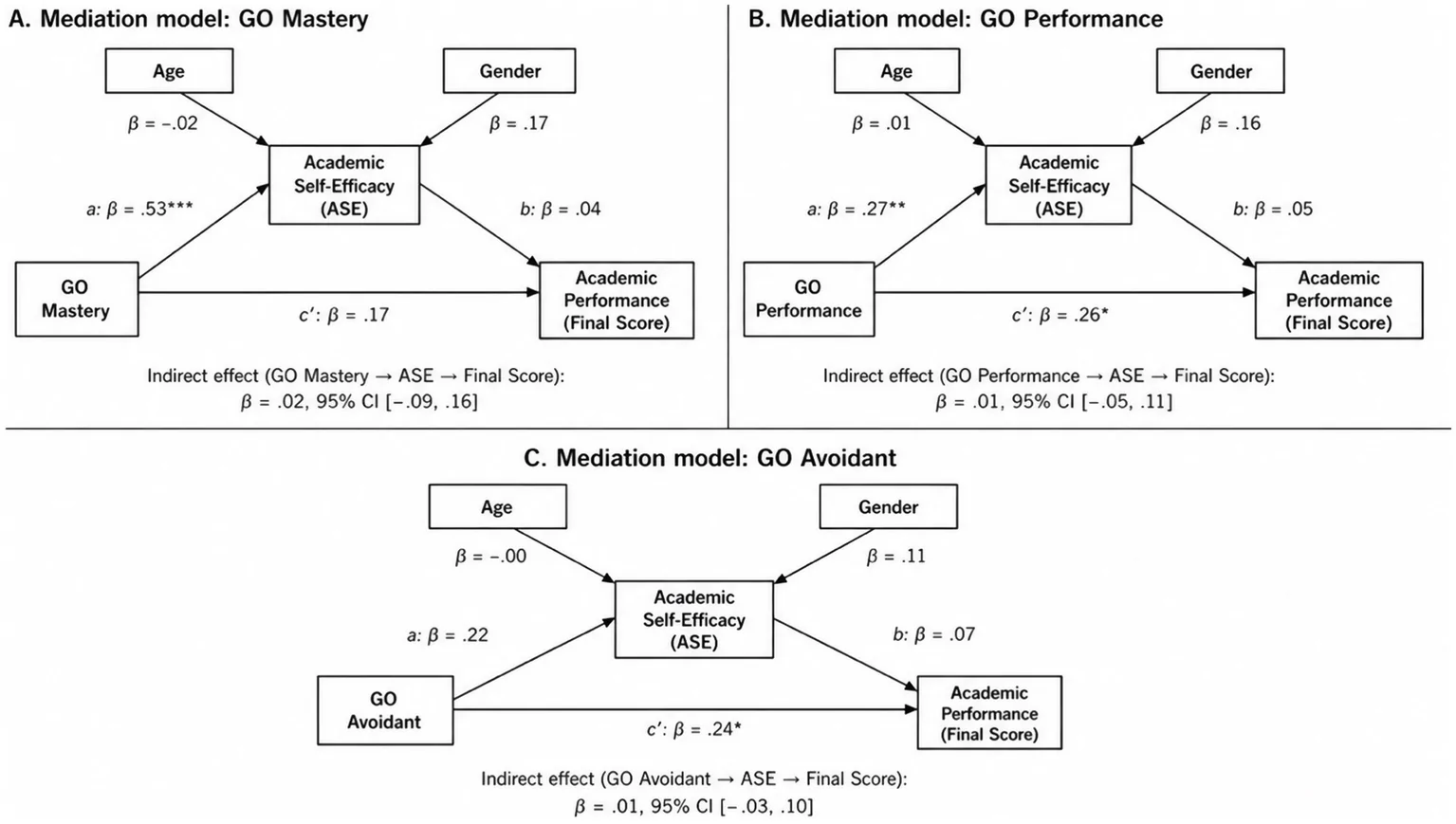
**(A)** Mediation model: GO Mastery; **(B)** Mediation model: GO Performance; **(C)** Mediation model: GO Avoidant. Standardized path coefficients (*β*) are shown. c', direct effect. *p < 0.05, **p < 0.01, ***p < 0.001. Cl, confidence interval (bias-corrected bootstrap, 5000 samples).

In the mastery GO model (Figure 2A), mastery GO was positively associated with academic self-efficacy (*β* = 0.53, *p* < 0.001), but was not significantly associated with academic performance (*β* = 0.17, *p* = 0.23). Academic self-efficacy was not significantly associated with academic performance (*β* = 0.04, *p* = 0.80).

In the performance GO model (Figure 2B), performance GO was positively associated with academic self-efficacy (*β* = 0.27, *p* = 0.008) and was also significantly associated with academic performance (*β* = 0.26, *p* = 0.02). Academic self-efficacy was not significantly associated with academic performance (*β* = 0.05, *p* = 0.69).

In the avoidant GO model (Figure 2C), avoidant GO was not significantly associated with academic self-efficacy (*β* = 0.22, *p* = 0.11), but was significantly associated with academic performance (*β* = 0.24, *p* = 0.02). Academic self-efficacy was not significantly associated with academic performance (*β* = 0.07, *p* = 0.59).

Across all models, age and gender were not significantly associated with academic self-efficacy or academic performance.

## Discussion

The current study examined how GO and academic SE related to academic performance in an applied, service-learning environment. The findings suggest that motivational relationships commonly observed in traditional academic settings may function differently in contexts that require interpersonal performance and real-time skill application. Specifically, performance GO and avoidant GO were positively associated with academic course performance, whereas mastery GO was not, and academic SE did not mediate these relationships (Table 3).

**Table 3:** Correlations between motivational variables and final score.

Variable	GO Mastery	GO Performance	GO Avoidant	Academic SE	Final score
GO Mastery	-				
GO Performance	0.16	-			
GO Avoidant	0.29**	0.59**	-		
Academic SE	0.52**	0.27*	0.22	-	
Final Score	0.21	0.30**	0.30**	0.10	-

GO, Goal Orientation represented by subscales mastery, performance, and avoidant. SE, self-efficacy. *p < 0.05, **p < 0.01, ***p < 0.001.

### Direct effects

One of the most notable findings was the positive association between avoidant GO and academic performance. Avoidant GO was positively correlated with final course performance (*r* = 0.30, *p* < 0.001), an association similar in magnitude to that observed for performance GO (*r* = 0.30, *p* < 0.001). The practically identical magnitude of these associations may suggest that performance and avoidant GO engage a shared motivational process within this service-learning context. Although the orientations reflect different achievement motives, both demonstrating competence and avoiding negative judgments may heighten responsiveness to performance demands when students’ skills are visible to others, potentially promoting increased preparation or effort. Avoidant GO is typically linked to maladaptive outcomes such as disengagement and lower achievement (Pintrich, 2000; Artino, 2012; Laitinen et al., 2024; Eum and Rice, 2011). The present findings therefore suggest that the relationship between avoidant GO and academic performance may be more context-dependent than traditionally assumed. In contexts where performance is socially evaluated and visible to others, such as client-facing environments, a desire to avoid negative judgment may motivate increased effort and engagement. This pattern does not necessarily indicate that avoidant orientation becomes inherently adaptive in service-learning contexts. Rather, the behavioral consequences of an avoidant orientation may depend on the demands and affordances of the learning environment (Pintrich, 2000). In settings requiring visible, real-time interpersonal performance, concern about negative evaluation may manifest as greater preparation, vigilance, or effort rather than disengagement, potentially producing short-term performance benefits even when the underlying motivational orientation remains avoidance-based.

However, greater effort or higher course performance should not necessarily be interpreted as evidence of higher-quality learning. Motivation driven by avoiding negative evaluation may support performance in the short term without promoting the deeper learning processes typically associated with mastery orientation, and its sustainability over time remains unclear. The current study assessed final course performance rather than learning depth or retention and therefore cannot determine whether the observed association with higher performance reflects higher-quality learning.

Social evaluation, perceived visibility of performance, and related contextual demands were not directly measured in the present study. Therefore, these factors should be considered potential explanations rather than established mechanisms underlying the observed association. Future research should directly assess these contextual dimensions to determine whether they influence the relationship between avoidant goal orientation and performance.

#### Mediation analysis

The mediation analyses did not support H2a-H2c, as academic self-efficacy did not significantly mediate the relationship between any GO subtype and academic performance. Notably, the indirect effects were nonsignificant across mastery, performance, and avoidant GO, indicating that academic self-efficacy did not explain the relationship between goal orientation and academic performance in this sample. This finding differs from prior research identifying self-efficacy as an important mechanism linking motivational orientations to academic achievement in traditional academic settings and suggests that this mediating pathway may not operate consistently across learning contexts (Honicke and Broadbent, 2016; Honicke et al., 2020).

The mastery GO findings provide additional insight into where the proposed theoretical pathway was not supported. Mastery GO was moderately associated with academic self-efficacy (*r* = 0.52, *p* < 0.001) but was only weakly and nonsignificantly associated with final course performance (*r* = 0.21, *p* > 0.05). This pattern was also evident in the mediation model, in which mastery GO significantly predicted academic self-efficacy, supporting the first component of the hypothesized pathway. However, academic self-efficacy did not significantly predict academic performance, indicating that the second component of the proposed pathway was not supported. Thus, the absence of mediation does not appear to reflect a lack of association between mastery orientation and perceived academic competence; rather, higher academic self-efficacy did not translate into higher final course performance in this sample. These findings provide support for the first component of the theoretical sequence underlying H2a but do not support academic SE as the mechanism through which mastery GO influences performance in this service-learning context.

The absence of a mediating effect of academic self-efficacy also raises the possibility that other contextual or interpersonal factors may contribute to performance in applied learning environments. For example, perceived social evaluation may influence students’ effort when their performance is visible to clients or instructors, whereas communication demands and the need to respond to clients in real time may involve competencies not captured by general academic self-efficacy. These factors were not directly measured in the present study and therefore should be considered potential rather than established mechanisms. Future research could directly assess perceived social evaluation, interpersonal or communication self-efficacy, and situational demands to determine whether these factors mediate or moderate relationships between goal orientation and performance.

#### Contributions

Together, these findings contribute to a more nuanced understanding of achievement motivation by suggesting that the relationships of GO and academic SE with performance may be more context-dependent than previously theorized. Traditional models of academic motivation, largely derived from classroom-based research, may not fully capture learning environments that emphasize applied performance and interpersonal interaction. In the present service-learning context, potentially relevant features included repeated client-facing interactions, visibility of students’ performance to others, the need to respond to clients in real time, and ongoing accountability for applying course-based skills across multiple coaching sessions. These features may increase the salience of competence demonstration and social evaluation and, in turn, influence how goal orientations are behaviorally expressed. Because these contextual dimensions were not directly measured, however, their role in explaining the observed relationships remains tentative and should be examined directly in future research.

#### Practical implications

These findings have implications for applied and experiential learning environments. The positive association between avoidant GO and academic performance suggests that motivation may be influenced by the evaluative and interpersonal demands of the learning context. In environments where performance is visible and socially evaluated, motivation driven by the desire to demonstrate competence or avoid negative judgment may facilitate greater engagement and effort.

For instructors designing service-learning or health professions education experiences, these findings suggest incorporating structured opportunities for students to practice skills under conditions that approximate the interpersonal demands of real-world performance. For example, instructors could use low-stakes simulated client interactions, peer practice, or formative skills demonstrations before students work directly with clients, coupled with specific feedback and opportunities to repeat or refine their performance. Clearly defined performance criteria and consistent rubrics may also help students understand expectations in socially evaluative settings. At the same time, because performance- and avoidant-oriented motivation may not necessarily promote deeper or sustained learning, instructors should pair performance-based assessment with opportunities for reflection, skill development, and improvement rather than relying primarily on social evaluation as a source of motivation.

More broadly, these findings highlight the importance of aligning evaluation structures with the interpersonal and applied demands of performance-based tasks while supporting students’ development of competence. Such approaches may allow instructors to use the authentic performance demands inherent in service-learning while providing opportunities for students to develop skills in a supportive and structured learning environment.

### Strengths and limitations

This study has several limitations that should be considered when interpreting the findings. First, the convenience sample was drawn from a single public health program, and the relatively small sample size may limit generalizability and statistical power, particularly for detecting indirect effects. Accordingly, the nonsignificant mediation effects should be interpreted cautiously and examined in larger, more diverse samples.

Second, academic self-efficacy and goal orientation were assessed only at baseline and may therefore primarily reflect pre-existing individual differences rather than motivational processes that developed during the service-learning experience. The study cannot determine whether these constructs changed throughout the course; future studies should assess them at multiple time points.

Third, the ASES-5 assesses general academic self-efficacy and may not fully capture self-efficacy specific to the interpersonal and applied competencies required in a service-learning environment. Future research should consider domain-specific measures that more closely align with the performance demands of applied learning contexts.

Prior coaching experience may also represent an unmeasured source of confounding. Although an exploratory item assessed previous coaching-related experience, variability in participants’ interpretation of the item prevented consistent classification of relevant coaching experience and, consequently, its inclusion as a covariate. Thus, the present analyses cannot determine whether prior coaching experience influenced the observed relationships among GO, academic SE, and academic performance. Future studies should use more clearly defined measures of prior coaching experience and examine its potential influence on these relationships.

Finally, although the applied and interpersonal nature of the service-learning environment was considered conceptually in the study design, specific contextual dimensions such as perceived social evaluation, visibility of performance, and interpersonal demands were not directly measured. Their proposed role in explaining the observed relationships therefore represents a post-hoc interpretation of the findings rather than a mechanism tested in the present study. These factors should consequently be considered tentative explanations, and future studies should directly assess these contextual dimensions to determine how features of applied learning environments influence motivational processes.

Despite these limitations, this study also has several notable strengths. It examines all three GO subtypes simultaneously, addressing a limitation in prior research that has often excluded avoidant orientation. Additionally, the study extends existing literature by investigating motivational processes within an applied, service-learning environment, providing insight into how GO and academic SE may function in contexts that differ from traditional classroom settings. These features contribute to a more comprehensive and context-sensitive understanding of achievement motivation.

## Data Availability

The raw data supporting the conclusions of this article will be made available by the authors, without undue reservation.
